# The fail-safe system to rescue the stalled ribosomes in *Escherichia coli*

**DOI:** 10.3389/fmicb.2014.00156

**Published:** 2014-04-10

**Authors:** Tatsuhiko Abo, Yuhei Chadani

**Affiliations:** ^1^Graduate School of Natural Science and Technology, Okayama UniversityOkayama, Japan; ^2^Department of Biology, Faculty of Science, Okayama UniversityOkayama, Japan

**Keywords:** ArfA, ArfB, ribosome rescue, *Escherichia coli*, *trans*-translation

## Abstract

Translation terminates at stop codon. Without stop codon, ribosome cannot terminate translation properly and reaches and stalls at the 3′-end of the mRNA lacking stop codon. Bacterial tmRNA-mediated *trans*-translation releases such stalled ribosome and targets the protein product to degradation by adding specific “degradation tag.” Recently two alternative ribosome rescue factors, ArfA (YhdL) and ArfB (YaeJ), have been found in *Escherichia coli*. These three ribosome rescue systems are different each other in terms of molecular mechanism of ribosome rescue and their activity, but they are mutually related and co-operate to maintain the translation system in shape. This suggests the biological significance of ribosome rescue.

## NON-PRODUCTIVE TRANSLATION COMPLEX FORMATION UPON TRANSLATION OF the mRNA LACKING STOP CODON AND ITS RESOLUTION BY *TRANS*-TRANSLATION

**FIGURE 1:**
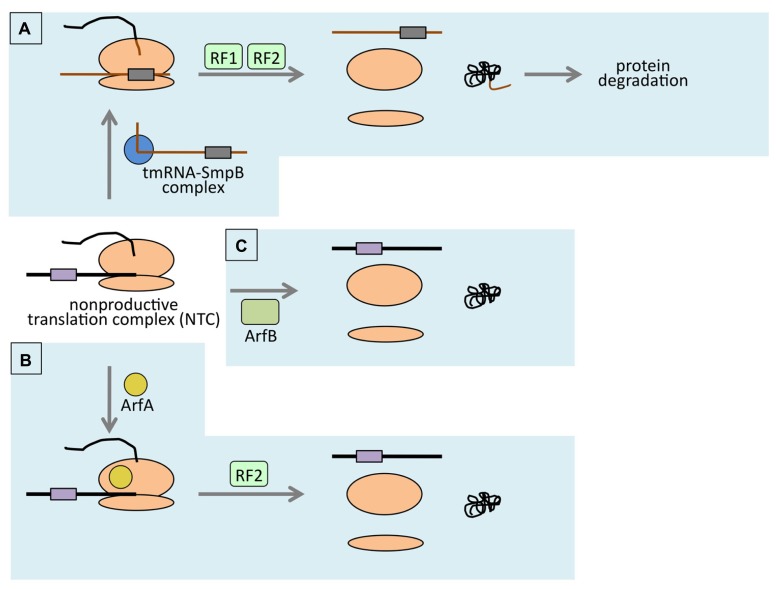
**Schematic representation of ribosome rescue in *E. coli*. (A)** tmRNA (brown line)- and SmpB (blue circle)-mediated *trans*-translation. In *trans*-translation, ribosome (orange ovals) switches its translating template from non-stop mRNA (black line) to tmRNA. Then it terminates translation at the stop codon (purple box) exist on the tmRNA. Protein product will be degraded due to the SsrA-tag (shown in brown) attached to the C-terminus. **(B)** ArfA-mediated ribosome rescue. RF2 is indispensable in this pathway. tRNA enhances this reaction by unknown mechanism. **(C)** ArfB-mediated ribosome rescue. As a stop codon-independent RF homolog, ArfB binds to A-site of the ribosome within NTC and hydrolyzes peptidyl-tRNA. Only the relevant factors are shown.

Translation is terminated when translating ribosome encounters in-frame stop codon. If mRNA lacks in-frame stop codon, translating ribosome cannot properly terminate translation. Such a ribosome reaches the 3′-end of mRNA and forms non-productive translation complex (NTC). Accumulation of NTC might be deleterious because it reduces active ribosomes which should be utilized in translation of other genes. To cope with this, bacterial cell is equipped with the ribosome rescue system which is called *trans*-translation (for reviews, see Moore and Sauer, 2007; Keiler, 2008; Hayes and Keiler, 2010; Barends et al., 2011; Janssen and Hayes, 2012; **Figure 1A**). As mentioned somewhere in this issue, *trans*-translation is the special type of translation mechanism occurs only when NTC is formed. The bifunctional tmRNA (SsrA RNA), which acts as both tRNA and mRNA, plays a key role in *trans*-translation and resolves the NTC with the assistance of specific partner protein SmpB (Keiler et al., 1996; Karzai et al., 1999). This enables the utilization of the ribosome for anther round of translation. At the same time, nascent polypeptide is tagged with tmRNA-specific peptide sequence, called SsrA-tag, at its C-terminus (Keiler et al., 1996). The polypeptide which has SsrA-tag at its C-terminus is lead to the active degradation by cellular proteases such as ClpXP, Lon, Tsp. Therefore, the SsrA-tag is also called degradation tag. The polypeptide encoded by non-stop mRNA is potentially harmful because it lacks the C-terminal region which may have important role in the function of the original protein. Considering this, addition of the SsrA-tag to the released polypeptide makes sense because it accomplishes the removal of potentially harmful truncated polypeptide.

*Trans*-translation also affects the fate of non-stop mRNA itself. Non-stop mRNA is deleterious to the cell and should be degraded as soon as it is produced for two reasons. First, it produces potentially harmful truncated polypeptide. Second, it traps ribosome and prevent further usage of the ribosome for another round of translation. On the other hand, NTC stabilizes non-stop mRNA by protecting it from the attack of 3′–5′ exoribonucleases, major ribonucleolytic factors in the bacterial cell. Thus, *trans*-translation also acts to prevent the accumulation of non-stop mRNA in the cells by resolving the NTC (Yamamoto et al., 2003).

Having three biologically important roles, ribosome rescue, protein quality control and mRNA quality control, *trans*-translation may be of great importance to bacterial cells. Adding to this, all the bacterial species whose genome were so far read have *ssrA* gene (Moore and Sauer, 2007). This strongly supports the importance of *trans*-translation.

## ALTERNATIVE PATHWAY FOR RIBOSOME RESCUE

Besides the proposed biological significance, tmRNA is dispensable in many bacterial species including *Escherichia coli* (Komine et al., 1994). This suggested the existence of another ribosome rescue system. The attempt to identify such system by genetic screening was difficult because it was “negative” screening. The first attempt to search for such factors was performed by Nakano et al. (2001). They employed penicillin resistance screening and isolated several mutants which caused the growth defect of the *E. coli* cells in the absent of *ssrA* gene. The genes isolated in their screening included *hisS*, *rho*, *prs*, and *degP* (*htrA*), but none of them had the function related to the ribosome rescue. Another screening was also attempted to fish out the gene that is required for the growth of *ssrA* mutant cell (Ono et al., 2009). In this case, the synthetic lethal screening originally developed by Bernhardt and de Boer (2004) which is based on the instability of the mini-F plasmid lacking *par* locus was employed. In the first trial, *degP* was identified as a candidate. But the DegP protease had nothing to do with ribosome rescue. Further analyses revealed that the lack of *trans*-translation somehow enhances the temperature sensitive (ts) phenotype of the *degP* mutant cell. This may involve the stress response pathway because the enhanced ts phenotype of *ssrA degP* double mutant cell was suppressed when RpoE activity was increased (Ono et al., 2009).

To look for the translation factors which show the phenotype in combination with *ssrA* mutation, the screening was repeated and a mutant whose growth was dependent on tmRNA has been obtained. This mutant cell had a base-substitution mutation in the *yhdL*, the open reading frame (ORF) of 72 codons whose function was not reported. The isolated A18T substitution of YhdL was loss-of-function mutation and the disruption of *yhdL* gene showed the same phenotype as A18T mutation. Genetic and biochemical studies which will be described below showed that YhdL has a ribosome rescuing activity and it was renamed ArfA for alternative ribosome rescue factor A (Chadani et al., 2010).

## *IN VIVO* EVIDENCES FOR ArfA-MEDIATED RIBOSOME RESCUE

Upon the simultaneous depletion of ArfA and tmRNA, *E. coli* cell stopped growing and this growth inhibition was correlated with the lowered level of translation (Chadani et al., 2010). SsrA^DD^, the tmRNA variant which attaches proteolysis-resistant mutant tag instead of degradation tag during *trans*-translation, is known to suppress the phenotype caused by the lack of tmRNA in many cases (Moore and Sauer, 2007; Keiler, 2008). This suggests that, of three proposed roles mentioned above, ribosome rescue is the most important role of *trans*-translation. SsrA^DD^ was shown to support the growth of the cell lacking both ArfA and tmRNA, strongly suggesting that ArfA is involved in ribosome rescue (Chadani et al., 2010). Activity of ArfA as a ribosome rescue factor was supported by the fact that the synthetic lethality of *arfA* and *ssrA* was suppressed in the presence of sub-lethal concentration of puromycin, the antibiotic reagent which inhibits translation. Puromycin accepts nascent polypeptide in the A-site of actively translating ribosome as an aminoacyl-tRNA mimic and irregularly terminates translation. As a result, translation complex composed of ribosome, mRNA, tRNAs, and other translation factors, is resolved. Suppression of the synthetic lethality of *arfA* and *ssrA* by puromycin was explained as a result of resolution of NTC formed at the 3′-end of non-stop mRNA (Chadani et al., 2010).

Direct evidence for ribosome rescue activity of ArfA was obtained by using artificially constructed model gene which has intrinsic transcription terminator within its ORF. It produces non-stop mRNA *in vivo* (Keiler et al., 1996). NTC formed at the 3′-end of such non-stop model mRNA is resolved by *trans*-translation system and the translation product (non-stop polypeptide hereafter) is lead to rapid degradation because of the degradation tag at its C-terminus. However, upon ArfA overexpression, non-stop polypeptide was detected. At the same time, non-stop mRNA level was reduced. Reduction of non-stop mRNA was explained as a result of the resolution of NTC which protected the non-stop mRNA from degradation by RNases. Conversely, in *arfA* background, the non-stop polypeptide was greatly reduced. These results suggested that ArfA rescues the ribosome stalled at the 3′-end of non-stop mRNA in the manner distinct from *trans*-translation (Chadani et al., 2010; **Figure 1B**).

## BIOCHEMICAL FEATURES OF ArfA

As a ribosome rescue factor, ArfA was likely to bind to ribosome and this was shown to be the case by several biochemical analyses. From the cell lysate, ribosome was pulled down with ArfA. Moreover, ArfA was detected in the ribosome-containing P100 fraction. These suggested that ArfA interacts with ribosome. Interestingly, A18T mutant of ArfA was also detected in P100 fraction, suggesting that A18T mutation has its effect on the function other than ribosome binding. Sucrose gradient analysis showed ArfA co-migrated with the large subunit of ribosome, suggesting that ArfA binds to the large subunit of ribosome (Chadani et al., 2010). Cross-linking experiment revealed the close proximity of ArfA and numbers of 50S ribosomal proteins, supporting the observation above. However, the positions of ribosomal proteins cross-linked with ArfA on the ribosome were relatively dispersed and the precise binding site of ArfA could not be determined (Chadani and Abo, unpublished result). High p*I* (predicted to be 10.29) of ArfA may contribute to the ribosome binding.

## *IN VITRO* ANALYSES OF RIBOSOME RESCUE

Because *arfA* and *ssrA* were synthetically lethal, ribosome rescue activity of ArfA could not be assessed further *in vivo*. Fortunately, this was accomplished using *in vitro* translation system. Hanes and Plückthun (1997) have reported that *trans*-translation activity in the S30 lysate can be blocked by oligodeoxyribonucleotide complementary to the part of tmRNA including its mRNA-like domain (MLD; anti-SsrA oligo). The situation of the double depletion of tmRNA and ArfA activities was achieved by adding anti-SsrA oligo to the S30 lysate prepared from *arfA*-defective *E. coli* cell (Chadani et al., 2010). NTC formed at the 3′-end of non-stop mRNA contains peptidyl-tRNA, and could be assessed by analyzing the peptidyl-tRNA in the translation reaction using neutral SDS-PAGE. Similar situation could be achieved by using reconstituted PUREsystem (Kuroha et al., 2009). Using these techniques, more detailed analyses of ribosome rescue were performed (see below).

## IDENTIFICATION OF THE THIRD RIBOSOME RESCUE FACTOR IN *E. coli*

Another ribosome rescue factor ArfB was isolated through the multicopy suppressor screening for *arfA ssrA* synthetic lethality. From this screening, *yaeJ* gene was found to support the growth of the *E. coli* cell simultaneously lacking *ssrA* and *arfA* genes, when its protein product was overexpressed from multicopy plasmid. This suggested that the YaeJ protein was the third ribosome-rescue factor. Indeed, analysis of *in vivo* expressed model non-stop construct revealed that YaeJ competed with tmRNA-mediated *trans*-translation upon overexpression as ArfA did. The YaeJ protein, when added to *in vitro* translation reaction lacking both *trans*-translation activity and ArfA activity, enhanced the release of nascent-peptide from NTC. Sucrose gradient analysis revealed that the YaeJ protein bound to the ribosome. The same analysis also showed that the peptidyl-tRNA disappeared from the ribosome-containing fraction and the mRNA in the same fraction was reduced, strongly indicating that YaeJ resolved NTC *in vitro*. Being the third ribosome rescue factor, *yaeJ* was renamed *arfB* for alternative ribosome rescue factor B (Chadani et al., 2011a; **Figure 1C**).

## STRUCTURAL FEATURES OF ArfB

The ArfB protein has been known to be a class I release factor homolog having a Gly-Gly-Gln (GGQ) motif, the critical motif for the hydrolytic cleavage of peptidyl-tRNA (Frolova et al., 1999; Scarlett et al., 2003; Youngman et al., 2008). Mutant ArfB which has Gly-Ala-Gln (GAQ) sequence instead of the GGQ motif retained the ribosome-binding activity, but did not enhance the cleavage of the bond between nascent peptide and tRNA of peptidyl-tRNA in S30 translation reaction (Chadani et al., 2011a; Handa et al., 2011). This strongly suggested that ArfB hydrolyzed peptidyl-tRNA within NTC depending the GGQ motif. ArfB may act as stop codon-independent RF homolog to rescue ribosome.

As class I release factor homolog, ArfB attracted the researchers’ interest, especially in structural biology field. The structure of the ArfB protein has been solved to be very similar to authentic class I release factors RF1 and RF2. Based on this observation, Handa et al. (2011) also showed its ribosome rescue activity. ArfB has long unstructured tail at its C-terminus. Truncated ArfB lacking its C-terminal tail could not support the growth of *arfA ssrA* double mutant cell, suggesting it is important for ArfB-mediated ribosome rescue. The fine structure of ArfB bound to the ribosome was revealed and it was suggested the C-terminal tail of ArfB lies in the mRNA path downstream of the 30S A-site (Gagnon et al., 2012). Mutational analysis revealed the important residues within the C-terminal tail of ArfB (Kogure et al., 2014).

The YaeJ homologs were also identified in human mitochondrial proteins. Among them was the ICT-1 protein, which closely linked to the human mitochondrial disease (Handa et al., 2010; Richter et al., 2010). *In vitro* analysis confirmed its ribosome-rescue activity (Richter et al., 2010). Existence of RF-homologs, presumably included in ribosome rescue, suggests the significance of ribosome rescue irrespective of the kingdom. There exist other RF homologs in mitochondria whose activity and function is still not known. It may be possible that the mitochondrial translation system has more than one ribosome rescue pathway.

## MOLECULAR MECHANISMS OF ArfA- AND ArfB-MEDIATED RIBOSOME RESCUE

Both ArfA and ArfB resolved the NTC formed at the 3′-end of artificially designed non-stop mRNA in the S30 lysate or the PUREsystem. However, when the NTC was isolated from translation reaction prior to ArfA- or ArfB-treatment, the result was different. ArfB could resolve the isolated NTC, whereas ArfA could not (Chadani et al., 2011a; Shimizu, 2012). Being RF homolog, ArfB might resolve the NTC by hydrolyzing peptidyl-tRNA in it (Chadani et al., 2011a; Handa et al., 2011). On the other hand, ArfA does not have GGQ motif which is the critical motif for hydrolysis of peptidyl-tRNA within ribosome. This lead to the assumption that ArfA required some factor(s) present in the S30 or PUREsystem translation reaction. To see what is required for ArfA-mediated ribosome rescue, each translation factor was checked for the ability to support ArfA-mediated resolution of the isolated NTC. By this simple screening, RF2 appeared to be the factor (Chadani et al., 2012; Shimizu, 2012). RF2 mutant whose GGQ motif was disrupted could not promote ArfA-pathway, suggesting that RF2 hydrolyzes peptidyl-tRNA with its GGQ motif in ArfA-mediated ribosome rescue. The amino acid residues directly involved in the stop codon recognition of RF2 was not critical for ArfA-mediated ribosome rescue (Chadani et al., 2012). It has been proposed that RF2 participates in ribosome rescue to some extent (Zaher and Green, 2009; Vivanco-Domínguez et al., 2012). ArfA may enhance this activity, presumably by recruiting RF2 to the A-site of stalled ribosome. Based on these observations, molecular mechanism of the ArfA-mediated ribosome rescue was illustrated as follows. First, ArfA somehow binds to the ribosome stalled at the 3′-end of non-stop mRNA. Then RF2 is recruited to the A-site of the stalled ribosome and hydrolyze the peptidyl-tRNA with its GGQ motif. How RF2 is recruited to the A-site of stalled ribosome by ArfA is still unclear. Interestingly, addition of tRNA molecules to the reaction enhanced ArfA- and RF2-mediated ribosome rescue activity. Considering that both tRNAs and class 1 RFs bind to the A-site to function, they may not function at the same time in ArfA-mediated ribosome rescue. It is much more probable that tRNA and RF2 functions sequentially. One possibility is that tRNAs somehow enhance the binding of ArfA to the A-site of the ribosome within the NTC, for example, by binding to the ribosome E-site (Chadani et al., 2012; Pech and Nierhaus, 2012).

*trans*-translation, ArfA pathway, and ArfB pathway have distinct target specificity, which may reflect the difference in the molecular mechanisms in rescuing ribosome. *trans*-translation occurs not only when ribosome stalled at the 3′-end of non-stop mRNA, but also when ribosome stalled within the mRNA (Janssen et al., 2013). Shimizu (2012) has shown that ArfA-mediated ribosome rescue needs unoccupied A-site, whereas ArfB can function on the stalled ribosome having mRNA in its A-site *in vitro*. This suggests that each ribosome rescue system has its own case to deal with, and they work together to keep the translation system in shape.

## *TRANS*-TRANSLATION-MEDIATED STRICT REGULATION OF ArfA EXPRESSION

**FIGURE 2:**
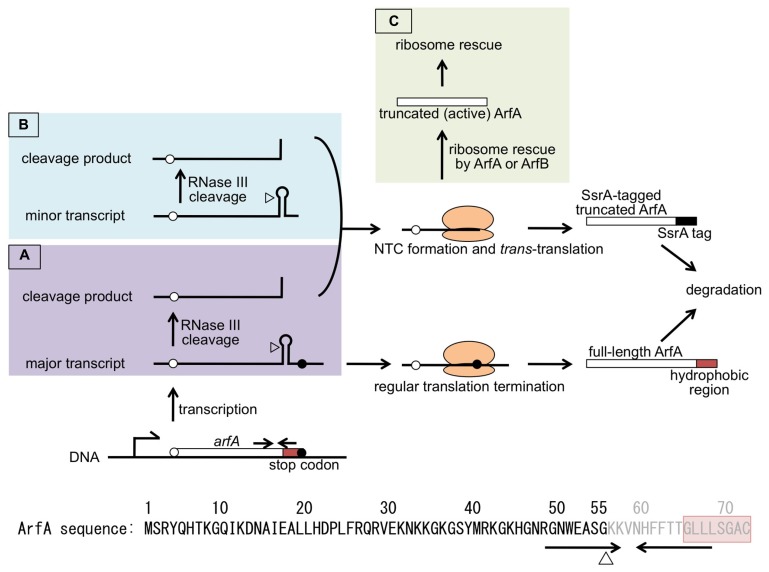
**Model for *trans*-translation-mediated regulation of ArfA expression.** *arfA* is transcribed as mRNA with **(A)** or without **(B)** stop codon. In both cases, mRNA is cleaved by RNase III at the site indicated by triangle. This results in the non-stop mRNA formation. ArfA translated from *arfA* mRNA with stop codon (**A**, bottom) will be degraded due to its hydrophobic C-terminus (red box). Translation of *arfA* non-stop mRNAs (**A**, top; **B**) results in NTC formation. The NTC is normally resolved by *trans*-translation and translated ArfA will be degraded due to the SsrA-tag (closed box) attached to its C-terminus. Once the level of NTC exceeds the capacity of *trans*-translation, NTC will be resolved first by SsrA-tagged ArfA escaped from proteolysis or ArfB, then by truncated ArfA which is produced from non-stop mRNA **(C)**. ArfA thus produced functions as a back-up ribosome rescue factor for *trans*-translation system. Amino acid sequence of ArfA is shown at the bottom. Positions corresponding to the inverted repeat (arrows) and RNase III cleavage site (triangle) are shown below the sequence. ArfA produced from the RNase III-processed *arfA* mRNA lacks its C-terminus portion which shown in gray characters.

ArfA is encoded as 72 a.a. protein on the *E. coli* genome (**Figure 2**). However, it was shown that ArfA was expressed from the non-stop mRNA. Nested deletion analysis showed that the 3′-region of *arfA* ORF had negative effect to the expression of *arfA* mRNA and ArfA protein (Chadani et al., 2011b). There were inverted repeat in the region and *arfA* mRNA could form stem-loop structure with some mismatches. This feature was commonly seen among the known RNase III cleavage sites (Régnier and Grunberg-Manago, 1990). When synonymous mutations which destabilize this stem-loop structure were introduced to the *arfA* ORF, level of *arfA* mRNA (named stem1 mutant) dramatically increased. Disruption of *rnc*, the gene coding for RNase III, gave a similar result. These strongly indicated that the stem-loop structure formed in the 3′-region was recognized and cleaved by RNase III, resulting in the formation of *arfA* non-stop mRNA (Chadani et al., 2011b; Garza-Sánchez et al., 2011). The RNase III cleavage site within *arfA* mRNA was mapped to the stem region by ligation-mediated RT-PCR.

Being non-stop mRNA, *arfA* mRNA is targeted by tmRNA-mediated *trans*-translation. This means that the protein product, C-terminally truncated ArfA, is SsrA-tagged and degraded in the cell. Indeed, truncated *arfA* mRNA and ArfA protein were increased in *ssrA* background. Moreover, in the presence of SsrA^DD^, DD-tagged truncated ArfA was detected. This indicated that ArfA expression was repressed post-transcriptionally by the mechanism involving *trans*-translation. Mass spectrum analysis has shown that the C-termini of DD-tagged and untagged ArfA proteins match the RNase III cleavage site (Chadani et al., 2011b).

Regulation of ArfA expression by *trans*-translation was shown to be strict. Even in the absence of RNase III, truncated *arfA* mRNA was detected. The 3′-end of this short mRNA was mapped just downstream of the inverted repeat. This was reasoned to be the result of premature transcription termination at the stem-loop structure. Adding to this, the full length ArfA protein produced from stem1 mutant *arfA* was unstable. Introducing two successive aspartates at the C-terminus of ArfA dramatically stabilized this protein. Considering that full-length ArfA has highly hydrophobic region at its C-terminus, it was suggested that the ArfA protein was degraded by the proteases which recognize hydrophobic C-terminal region (Parsell et al., 1990; Chadani et al., 2011b).

From these results, the strict regulation of ArfA by the mechanism containing non-stop mRNA production and *trans*-translation-mediated protein degradation was illustrated (**Figure 2**). Regardless of premature transcription termination, *arfA* transcripts are cleaved by RNase III at the stem-loop structure. This cleavage makes *arfA* mRNA non-stop which would be targeted by *trans*-translation. This results in shutting out of ArfA expression. Some part of *arfA* transcripts may escape from both premature termination and RNase III cleavage. But the resulting “full-length” ArfA is susceptible to the degradation by cellular proteases. So, if *trans*-translation is active enough, ArfA expression is strictly repressed. On the other hand, if the overall non-stop mRNA is beyond the capacity of *trans*-translation, certain amount of the NTC formed at the 3′-end of *arfA* non-stop mRNA would be left unresolved. Such NTC might be rescued by ArfA which escaped from proteolysis or ArfB, both of which rescues stalled ribosome without leading the nascent truncated polypeptides to degradation. C-terminally truncated ArfA is active in terms of ribosome rescue activity. Once produced, truncated but active ArfA rescues stalled ribosomes. Such ribosomes include that stalled at *arfA* non-stop mRNA. As a result, ArfA level would rapidly increase and the NTCs accumulated in the cell would be resolved. This novel strict regulation system involving *trans*-translation is rational for the biological role of ArfA as a back-up ribosome rescue system. Moreover, *arfA* represents the first gene shown to be expressed as a non-stop mRNA in regular state and regulated strictly by *trans*-translation. It has been reported that *Bacillus subtilis* KinA is also expressed as non-stop mRNA, but it was because of the accidental recombination (Kobayashi et al., 2008).

## CONCLUSION: SIGNIFICANCE OF RIBOSOME RESCUE IN *E. coli*

**FIGURE 3:**
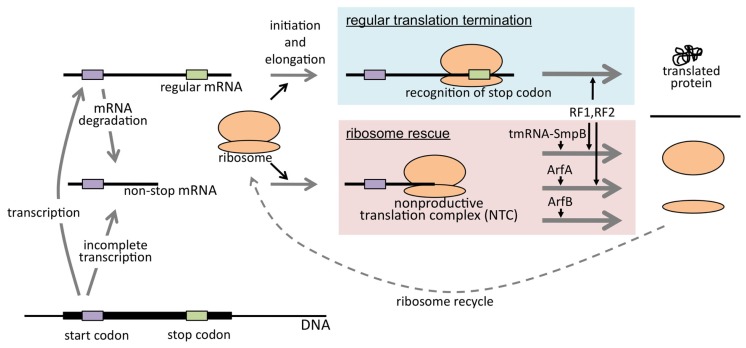
**Biological significance of ribosome rescue.** Non-stop mRNA is produced either by immature transcription or degradation of mature mRNA and causes NTC formation when translated. *E. coli* cell is equipped with at least three ribosome rescue systems mediated by tmRNA–SmpB complex, ArfA, or ArfB. In either case, NTC is resolved and rescued ribosome can be utilized in another round of translation.

It has been shown that *E. coli* cells are equipped with at least three ribosome rescue systems. This suggests that the accumulation of NTC is deleterious to the cells. The three systems look different in their role in ribosome rescue. According to the model, tmRNA-mediated *trans*-translation is a major ribosome rescue system (**Figure 2**). As mentioned above, this system simultaneously rescues the ribosome and leads the nascent polypeptides to degradation. Though latter seems not as important as former property, as judged by the fact that SsrA^DD^ can support the growth of the cell, degradation of potentially harmful truncated polypeptides may have some advantage (see below). Once the level of non-stop mRNA exceeds the capacity of *trans*-translation, another ribosome rescue factor ArfA is expressed and ArfB-mediated ribosome rescue may be required for early stage of ArfA expression. These three systems may cooperate as a fail-safe mechanism to maintain the sound translation system (**Figure 3**).

Though *ssrA*-deficient *E. coli* cell has been shown to have several phenotypes, it is basically viable, except for the extreme situations such as high temperature (Komine et al., 1994), presence of several antibiotics (Abo et al., 2002; Luidalepp et al., 2005) or amino-acid starvation (Li et al., 2008). On the other hand, it is known that *trans*-translation-mediated ribosome rescue is essential in *Neisseria gonorrhoeae* (Huang et al., 2000). Recently, *Shigella flexneri* has been also shown to require tmRNA (Ramadoss et al., 2013). Blast search showed these bacteria do not have the protein which shows significant homology with *E. coli* ArfA (Chadani et al., 2010; Ramadoss et al., 2013). Lack of the alternative ribosome rescue system may be the reason why *trans*-translation system is essential for them. The lethal phenotype of tmRNA depletion in *N. gonorrhoeae* and *S. flexneri* were suppressed by SsrA^DD^ and *E. coli* ArfA, respectively, suggesting the importance of ribosome rescue in bacteria.

As mentioned above, *ssrA*-deficient *E. coli* cell shows ts phenotype (Komine et al., 1994). Adding to this, disruption of *ssrA* enhances the ts phenotype of *degP*-deficient *E. coli* cell (Ono et al., 2009). This enhanced ts phenotype was suppressed by the overexpression of RpoE, the stress-response sigma factor. ArfA was present in both cases and SsrA^DD^ could not suppress the ts phenotype of the *ssrA degP* double mutant (Ono et al., 2009). This suggests that protein quality control at elevated temperature is critical to bacterial cells. This fits the proposed role of ArfA as a back-up system for *trans*-translation, which has both protein quality control and ribosome rescue functions.

Ribosome stalling occurs not only at the 3′-end of non-stop mRNA but also within ORF (for recent review, see Giudice and Gillet, 2013). In some cases, stalled ribosomes would resume the translation by the pathway involving the factors such as EF4 (Pech et al., 2011) or EF-P (Doerfel et al., 2013; Ude et al., 2013). Alternatively, they will be released by spontaneous drop-off from the mRNA or subjected to A-site cleavage by nucleases such as RelE (Christensen and Gerdes, 2003), MazF (Christensen et al., 2003), or RNase II (Garza-Sánchez et al., 2009). In former case, peptidyl-tRNA will be resolved by peptidyl-tRNA hydrolase (Pth; Singh et al., 2008). In latter case, ribosomes will be rescued by *trans*-translation, ArfA pathway, or ArfB pathway. It is interesting if deficiency or dysfunction of these factors have something to do with the reported SsrA-depletion-related ts phenotype of *E. coli* or lethal phenotype of *N. gonorrhoeae*.

There are several problems to be answered. The molecular mechanism of ArfA- and RF2-mediated ribosome rescue is still unknown. Structural analysis of ArfA might shed light to this. Also, ArfA is distributed only in *E. coli* and closely related bacteria. Whether similar mechanisms exist in other species or not is another important question.

## Conflict of Interest Statement

The authors declare that the research was conducted in the absence of any commercial or financial relationships that could be construed as a potential conflict of interest.
